# Supplementary material for: Deciphering the cellular tumor microenvironment landscape in salivary gland carcinomas using multiplexed imaging mass cytometry
Source: J Exp Clin Cancer Res. 2025 Oct 13;44:288. doi: 10.1186/s13046-025-03551-z (PMC12516863; doi:10.1186/s13046-025-03551-z)

Supplementary Figure S1

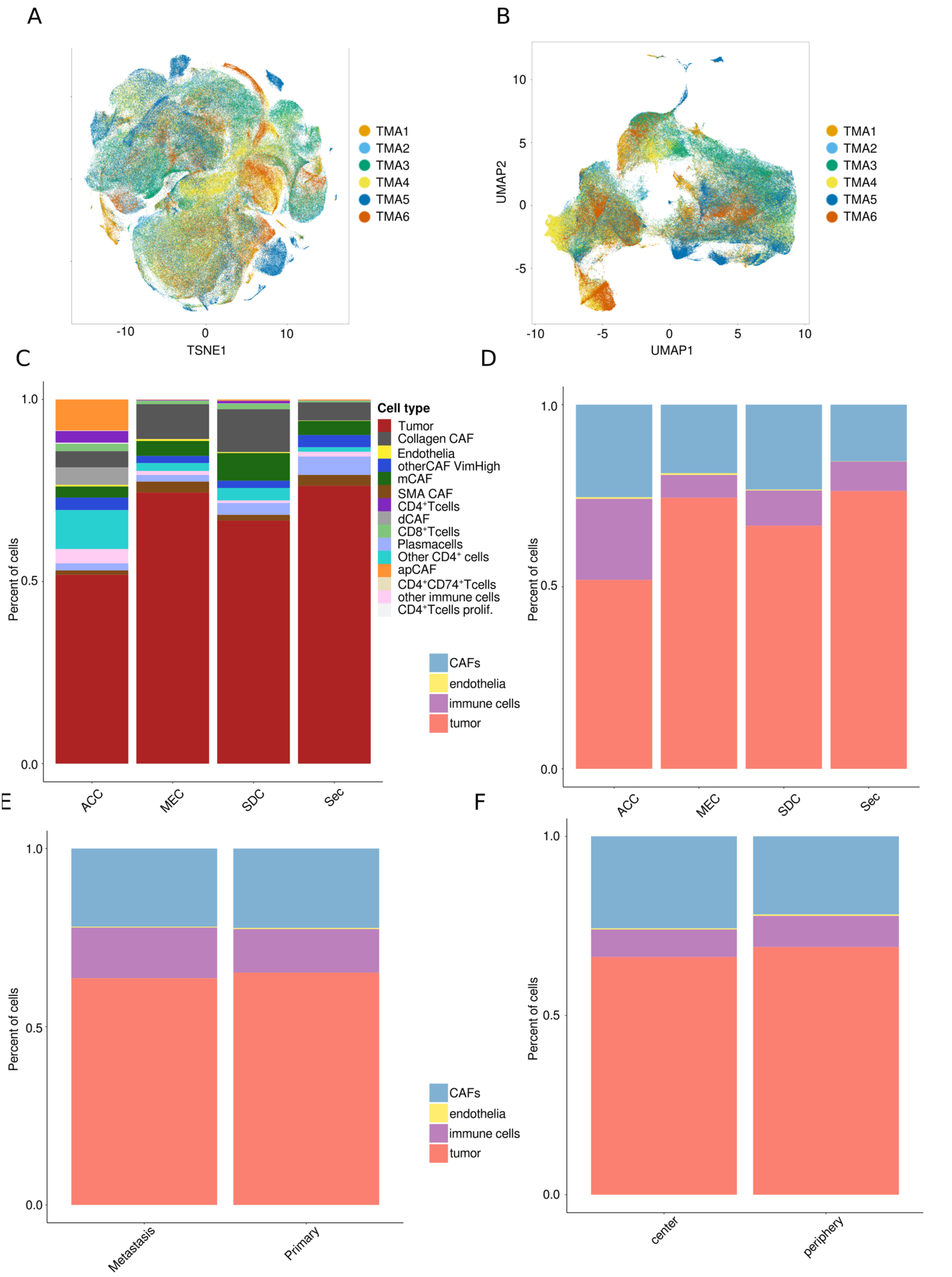

Supplementary Figure S2

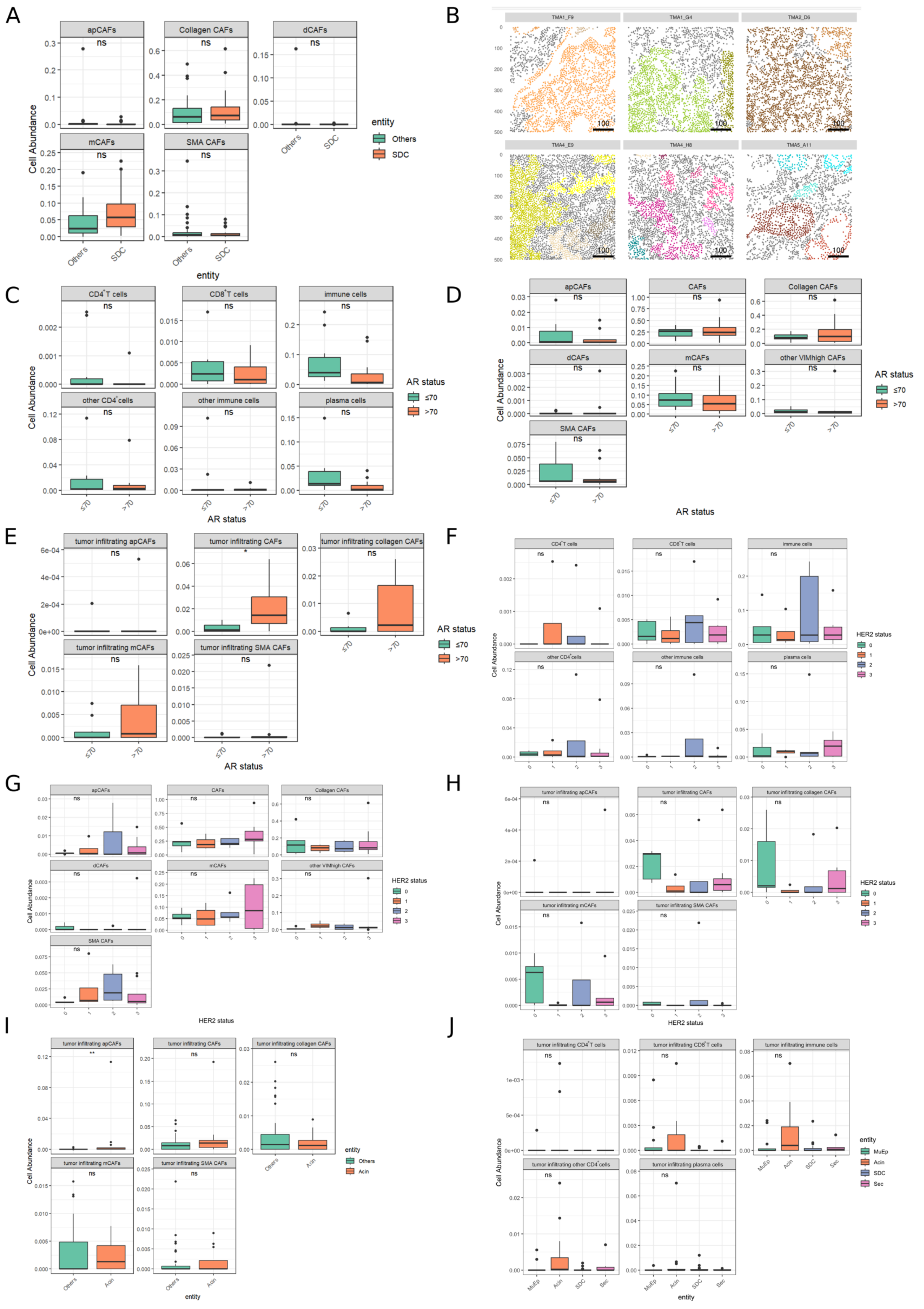

Supplementary Figure S3

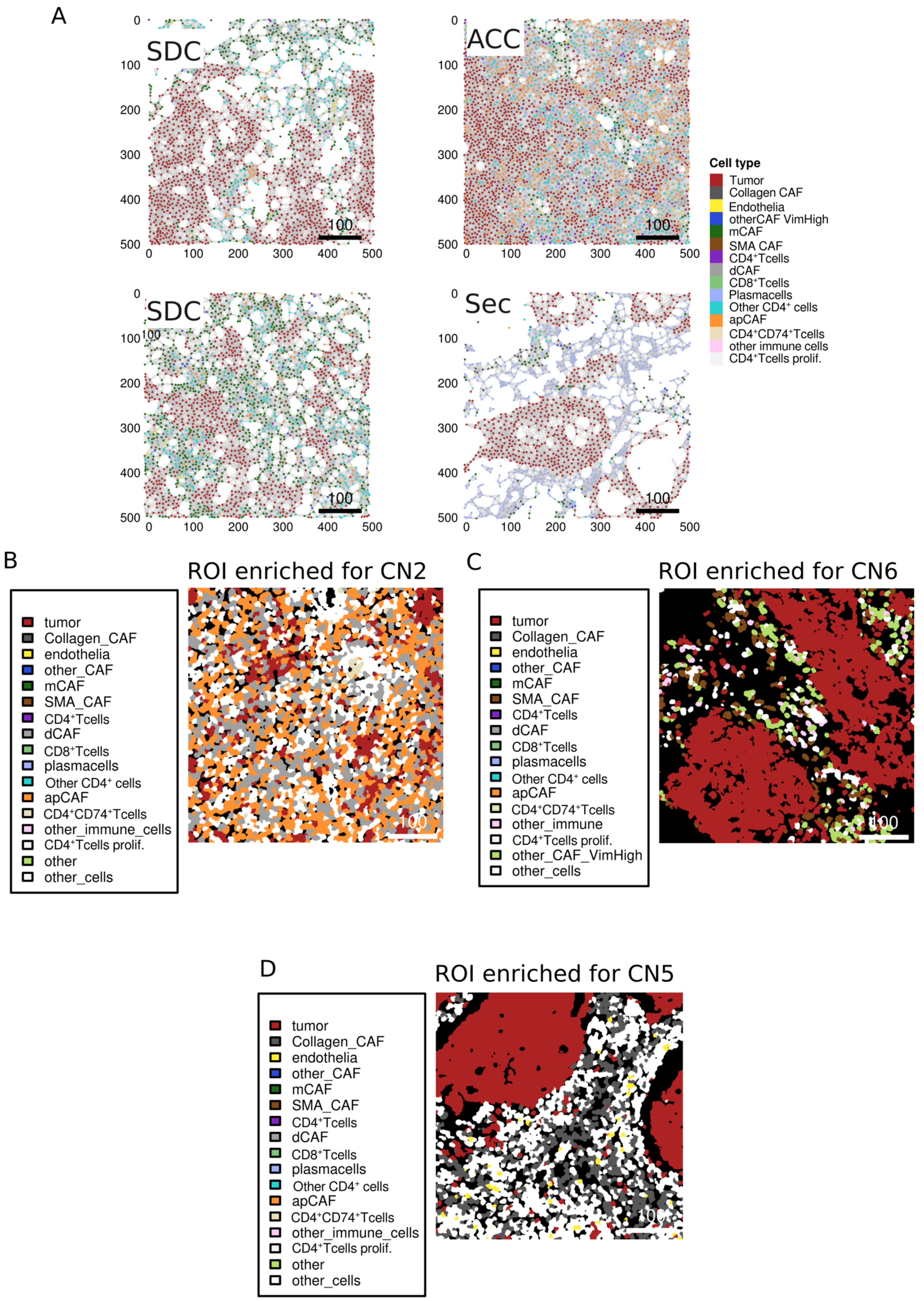

Supplementary Figure S4

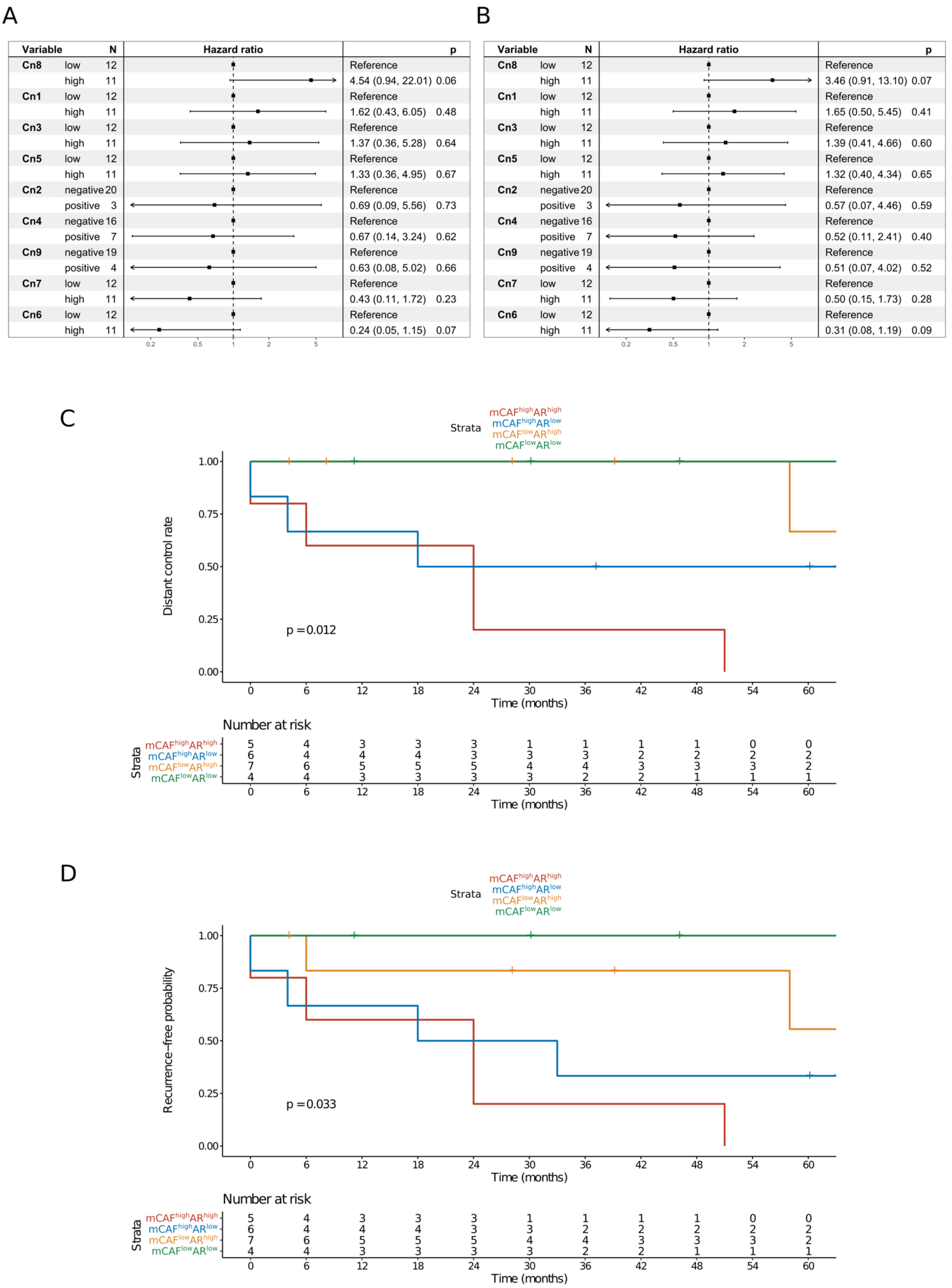

Supplementary Figure S5

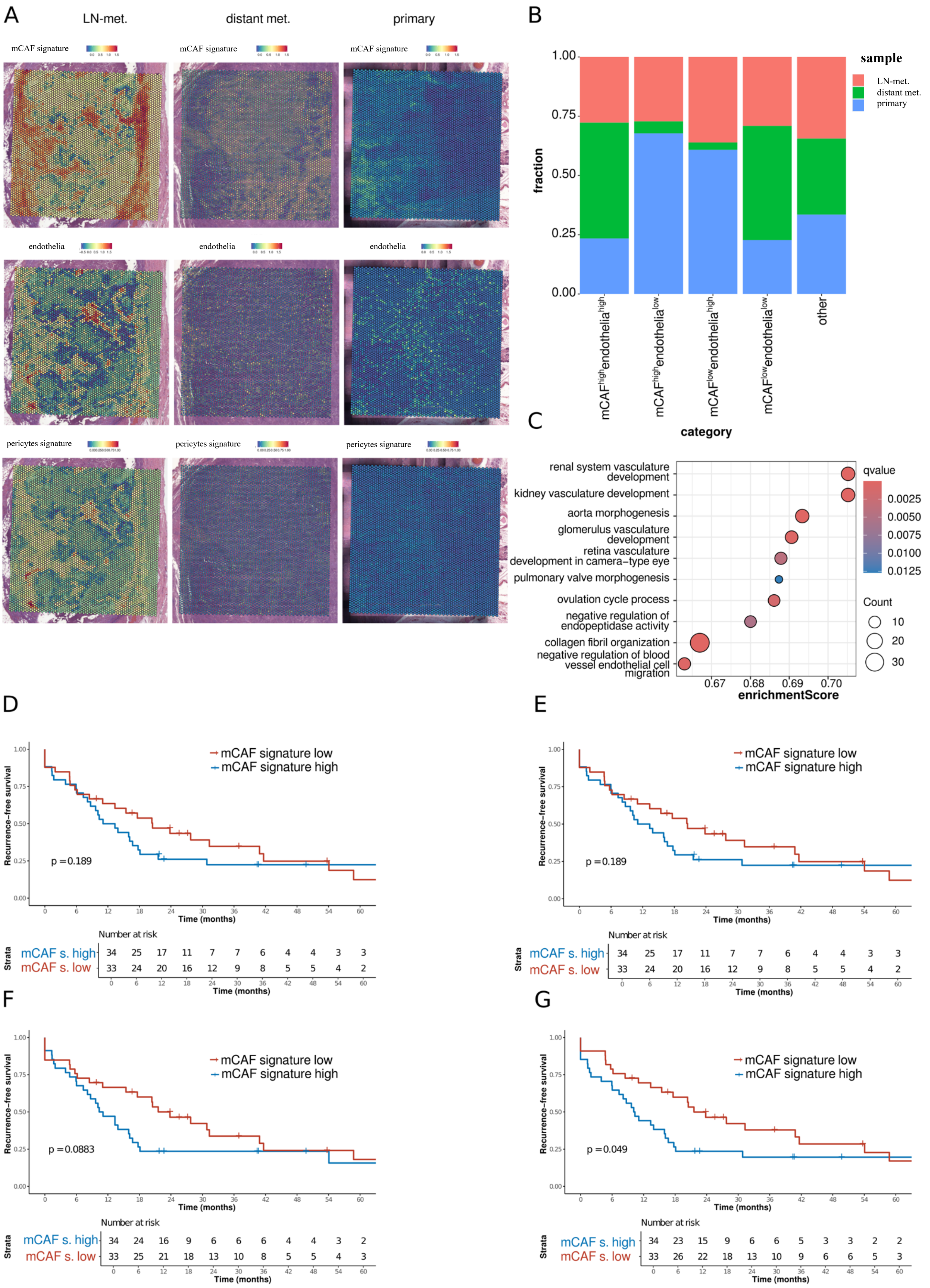

Supplementary Figure S6

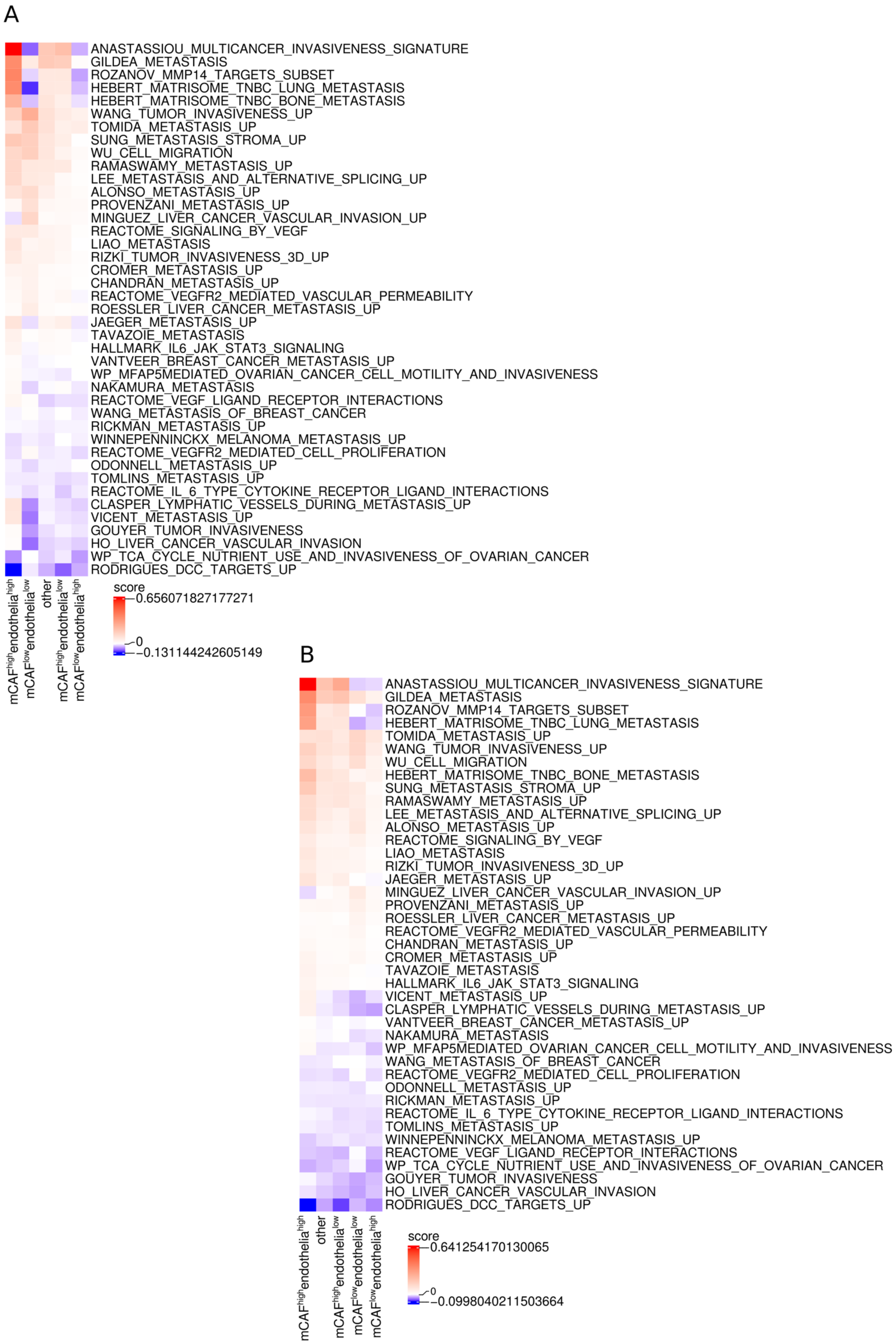

Supplement: Supplementary file 1 — Supplementary Material 1: Supplementary Figure S1: (A) tSNE and (B) UMAP projection of all analyzed cells colored by TMA. (C) Overall cell type and (D) cell category frequency. (E) Cell category frequency of primaries as well as metastases and (F) central and tumor compartments of the primary (all entities pooled). Supplementary Figure S2: (A) Abundance of CAF subsets in SDC versus other tumor types. (B) Examples of tumor patch detection (tumor patches are colored, other cells depicted in grey). (C-E) Frequency of immune cells, CAFs and tumor-infiltrating CAFs compared between ARhigh and ARlow SDC. (F-I) and between SDC with different HER2 expression levels. (J) Frequency of tumor-infiltrating immune cells in SGC entities. Supplementary Figure S3: (A) Examples of cellular interaction graphs via k nearest neighbor detection with k = 20; colors correspond to cell types. (B-D) exemplary ROIs with co-localization of cells that are characteristic for cellular neighborhoods 2, 6, and 5. 100-micron scale bars. Supplementary Figure S4: Univariate Cox proportional hazards model for (A) DCR and (B) RFP for cluster numbers (Cn) stratified by median proportion or as negative vs. positive in case median equals zero. Kaplan-Meier plots for C) DCR and (D) RFP with log-rank tests comparing patients with high and low mCAF frequencies stratified by AR status (ARhigh >70% and ARlow ≤70 of tumor cells positive for AR). Supplementary Figure S5: A) Spatial expression of the mCAF, pericytes, and endothelia module scores. B) Fraction of spots of each ST-sample within each spot category as defined by expression of the module scores for mCAFs and endothelia. C) Gene set enrichment analysis of DE-results depicted in Figure 5 C using “Biological Processes” GO terms. D-G) Estimation of recurrence-free survival in 67 SDC patients which were median-dichotomized using mCAF signatures derived with different log2FC cutoffs (Cords et al., Nat Com., 2023): (D) top 5 genes, (E) top 5%, (F) top 10% and [file 13046_2025_3551_MOESM1_ESM.pdf]
